# Structural basis of empathy and the domain general region in the anterior insular cortex

**DOI:** 10.3389/fnhum.2013.00177

**Published:** 2013-05-09

**Authors:** Isabella Mutschler, Céline Reinbold, Johanna Wankerl, Erich Seifritz, Tonio Ball

**Affiliations:** ^1^Department of Psychology, Division of Clinical Psychology and Epidemiology, University of BaselBasel, Switzerland; ^2^Department of Psychiatry, University of California San Diego (UCSD)La Jolla, California, USA; ^3^Department of Psychology, Division of Molecular Neuroscience, University of BaselBasel, Switzerland; ^4^Department of Psychiatry, Psychotherapy and Psychosomatics, Zurich University Hospital of PsychiatryZürich, Switzerland; ^5^Intracranial EEG and Brain Imaging Research Group, University of FreiburgFreiburg, Germany

**Keywords:** social neuroscience, individual differences, emotion, pain, sensorimotor integration, auditory perception, language, voxel-based morphometry

## Abstract

Empathy is key for healthy social functioning and individual differences in empathy have strong implications for manifold domains of social behavior. Empathy comprises of emotional and cognitive components and may also be closely linked to sensorimotor processes, which go along with the motivation and behavior to respond compassionately to another person's feelings. There is growing evidence for local plastic change in the structure of the healthy adult human brain in response to environmental demands or intrinsic factors. Here we have investigated changes in brain structure resulting from or predisposing to empathy. Structural MRI data of 101 healthy adult females was analyzed. Empathy in fictitious as well as real-life situations was assessed using a validated self-evaluation measure. Furthermore, empathy-related structural effects were also put into the context of a functional map of the anterior insular cortex (AIC) determined by activation likelihood estimate (ALE) meta-analysis of previous functional imaging studies. We found that gray matter (GM) density in the left dorsal AIC correlates with empathy and that this area overlaps with the domain general region (DGR) of the anterior insula that is situated in-between functional systems involved in emotion–cognition, pain, and motor tasks as determined by our meta-analysis. Thus, we propose that this insular region where we find structural differences depending on individual empathy may play a crucial role in modulating the efficiency of neural integration underlying emotional, cognitive, and sensorimotor information which is essential for global empathy.

## Introduction

Empathy has strong implications for manifold domains of social behavior and it may constitute an integral part of emotional intelligence (Goleman, 1998). In the psychological literature, empathy has been defined as the ability to feel what another person is feeling (emotional component), and knowing what a person is feeling (cognitive component), i.e., to achieve a cognitive understanding of other feelings (Eisenberg and Miller, 1987; Decety and Jackson, 2004). Empathy may also include sensorimotor processes, which go along with the motivation and behavior to respond compassionately to another person's feelings (Preston, 2007; Zaki and Ochsner, 2012). It has been shown that empathetic ability, however, is not always going along with prosocial behavior (Eisenberg and Miller, 1987). The cognitive component of empathy may be closely linked to “theory of mind,” that is the meta-cognitive ability to represent mental states such as beliefs, intentions, and desires of other people (Premack and Woodruf, 1978). According to Dymond, an empathic person can imaginatively take the role of another and can understand and predict that person's thoughts, feelings, and actions (Dymond, 1949). Together, these definitions suggest that the human ability of empathy is more than a pure emotional process but also includes cognitive aspects such as perspective taking and may also involve the sensorimotor system for responding appropriately and compassionately to another person's feelings. There is growing appreciation that there are stable differences between individuals in the level of empathy which has a high impact on social behavior (Zaki and Ochsner, 2012) and that these differences can be reliably measured (Miller and Eisenberg, 1988; Marangoni et al., 1995; Singer et al., 2004). Recent studies have shown that there is capacity for local plastic change in the structure of the healthy adult human brain in response to environmental demands or intrinsic factors (Johansen-Berg, 2012). There is increasing interest in investigating the neuroanatomical basis underlying individual differences in human behavior and cognition (Kanai and Rees, 2011), including empathy (Banissy et al., 2012). However, the structural basis of empathy in particular in the insular cortex has remained unclear. Accumulating evidence indicates a crucial role of the insular cortex in empathy: in particular the anterior insular cortex (AIC)—a brain region which is situated in the depth of the Sylvian fissure and anatomically highly interconnected to many other cortical regions (Nieuwenhuys, 2012)—is part of the functional neural network that plays an essential role in mediating social-emotional processing (Mutschler et al., 2009, 2012) including empathy (Singer et al., 2004; Seeley et al., 2012).

Deficits in empathy have been reported in different neuropsychiatric conditions such as in borderline personality disorder with a history of childhood trauma and co-morbid posttraumatic stress disorder (Roepke et al., 2012), autistic spectrum disorders (Decety and Moriguchi, 2007), in psychopathy/antisocial personality disorder (Decety and Moriguchi, 2007; Shirtcliff et al., 2009), in conduct disorder (Sterzer et al., 2007), and in schizophrenia (Bora et al., 2008). In several of these disorders structural abnormities in the anterior insula have been shown such as in adolescents with conduct disorder (Sterzer et al., 2007), in adults with schizophrenia (Makris et al., 2006), and in individuals with psychopathy (de Oliveira-Souza et al., 2008). More recently, a study in combat veterans with traumatic brain injury reports that lesions in several brain regions, particularly in the insula, was associated with deficits in empathy (Driscoll et al., 2012).

On this background we used voxel-based morphometric (VBM) analysis of high-resolution structural MRI to investigate the correlation between local gray matter (GM) density and inter-individual differences in empathy in a large sample of healthy adult females (*n* = 101). VBM allows objective structural analysis across the whole brain in an unbiased way and with no *a priori* regions-of-interest (ROIs). A purely female sample was investigated because there is increasing evidence for sex differences in empathy (Hoffman, 1977; Baron-Cohen and Wheelwright, 2004).

Because of the increasing evidence indicating a crucial role of the insular cortex in empathy a further aim of this study was to put empathy-related structural effects into the context of a functional map of the AIC determined by activation likelihood estimate (ALE) meta-analysis of previous functional imaging studies. While the anatomical diversity of AIC is long recognized (Mesulam and Mufson, 1982; Mufson and Mesulam, 1982), functional studies have often treated the AIC as a single, homogenous region. Recently, however, a differentiated functional map of the AIC in the human brain is emerging, primarily based on meta-analytic summaries of neuroimaging studies (Mutschler et al., 2007, 2009, 2012) and connectivity studies (Cauda et al., 2011; Deen et al., 2011; Chang et al., 2013; Touroutoglou et al., 2012). These studies together clearly indicate that the AIC contains several functionally specialized parts, related to pain experience, auditory processing and language, and sensorimotor functions, among others. An further functional subregion in the AIC is the *domain general region* (DGR) (Dosenbach et al., 2006). It is outstanding that this region is activated across a broad range of tasks typically investigated in functional imaging studies (Craig, 2009) and has been proposed that it represents a potential high-level insular integration hub possibly involved in task-set representation (Dosenbach et al., 2006), awareness (Craig, 2009), rule-based evaluation of sensory information (Mutschler et al., 2009), and evaluation of the salience of (internal and external) sensory information (Wiech et al., 2010; Mutschler et al., 2012; Borsook et al., 2013).

Because empathy also crucially requires high-level integration of emotional, cognitive, and social components as well as of behavioral control, we were specifically interested whether there are structural differences in the insular DRG related to individual empathy and more generally, how empathy-related structural effects map onto functionally defined insular areas related to pain-processing, emotion, and sensorimotor functions. To this end, in the present study VBM findings were related to a recently developed functional map of the AIC and this map was extended to by meta-analysis of previous neuroimaging studies investigating empathy for pain and emotional processing applying the ALE method (Turkeltaub et al., 2002).

## Materials and methods

### Subjects

One hundred and one healthy females (mean age = 23.6 years, range = 18–35 years) with no history of psychiatric or neurological diseases (based on a clinical interview by a trained psychologist) were recruited from the local University, ensuring a comparable/similar educational background. All participants were right-handed according to the Edinburgh Handedness Questionnaire (Oldfield, 1971): mean = 83.7%, range = 58.3–100%. This study was approved by the local ethics committee for medical research in Basel. Before participation, subjects signed written informed consent. They received a modest monetary compensation for participation.

### Data acquisition

T1-weighted high-resolution images were collected with a 3 T scanner (Siemens Magnetom Allegra syngo MR 2004A, Erlangen, Germany) using a MPRAGE sequence (resolution: 1 mm isotropic, matrix: 256 × 256 × 176, TR: 2000 ms, TI: 1000 ms, 7° flip angle). There were no structural abnormalities in any of the MRIs. To measure individual differences in trait empathy we used the E-Scale, a commonly used and validated 25-item self-evaluation measure by (Leibetseder et al., 2001). Twenty-two of the items are worded positively so that agreement indicates higher empathy. The other three items are worded negatively so that disagreement indicates higher empathy. The technique of balancing positively and negatively worded items helps to control for acquiescence bias. Thirteen items assess empathy in fictitious and 12 items empathy in real-life situations. Each items has to be answered on a 7-point Likert scale. The total score is computed by adding up the participant's responses to the 22 positively and 3 negatively worded items. This German version has been developed from English instruments assessing empathy (Mehrabian and Epstein, 1972; Stotland et al., 1978; Davis, 1983). For a more detailed discussion of the psychometric properties of the measure and how it was developed see reference Leibetseder et al. (2001). A more recent study shows that the E-Scale assesses cognitive and emotional components of empathy and shows that this measure possesses high reliability and validity for the assessment of trait empathy (Leibetseder et al., 2007).

### Voxel-based morphometry (VBM) analysis

DARTEL-based VBM analyses of combined gray-white maps were performed on the structural MRIs. Data were processed using the VBM8 toolbox (http://dbm.neuro.uni-jena.de/vbm8/) and the SPM8 (http://www.fil.ion.ucl.ac.uk/spm/) software package. The images were segmented into GM, white matter (WM) using SPM8, and then normalized with the Dartel normalization, using the VBM8 Dartel template (Ashburner, 2007), including a modulation step. A manual quality control and inspection of the processing steps and outputs was performed. Finally, normalized images were smoothed with a 6-mm full-width at half-maximum (FWHM) Gaussian kernel. Statistical analysis was carried out by means of voxelwise whole brain correlation of MR signal intensities with the individuals' empathy scores. Similar to several previous VBM studies (Kim et al., 2008; Labate et al., 2008; Peters et al., 2009; Heuser et al., 2011; Herringa et al., 2012), we report our results at *p* < 0.001, uncorrected, combined with a conservative threshold on cluster size, requiring more than 150 contiguous significant voxels (*k* > 150). Normal distribution of the questionnaire data was tested with the Kolmogorov–Smirnov test, and analyzed using SPSS (Version 19.0, Chicago, IL, USA).

### Activation likelihood estimate (ALE) meta-analysis

#### Search criteria

A search of Medline (Medical Literature Analysis and Retrieval System Online) and PsycINFO (Psychological Information Database) was performed. No start data limit on the search criteria if the databases were set but the end date was end of December 2011. The search keywords in title and abstracts were “empathy,” “pain,” “noxious stimuli,” “emotion,” “brain imaging,” “insula,” “insular cortex,” “functional magnetic resonance imaging” (fMRI”), “positron emission tomography” (“PET”).

#### Inclusion criteria

(1) The article had to be published in a peer-reviewed journal; (2) The study investigated empathy for pain, or emotional processing or experimentally induced physical pain in individuals using fMRI or PET; (3) Reported insular peaks that lay within 5 mm of the insular border (for more details see below); (4) Provided Talairach or Montreal Neurological Institute (MNI) coordinates; (5) Included hand movement (e.g., button press) only if two experimental conditions assumed to have equal movement-related activity were contrasted; this was selected to rule out movement-related effects, which are known to activate a region in the AIC (Mutschler et al., 2007, 2009); (6) Examined healthy adult individuals that did not suffer from any neurological or psychiatric disorder. The studies included in the ALE analyses according to these inclusion criteria are listed in the Appendix.

#### Analyses

Talairach coordinates were translated to the MNI space using the tal2mni Matlab script from http://eeg.sourceforge.net/doc_m2html/bioelectromagnetism/tal2mni.html. Subsequently, an ALE, given by the union of the probabilities associated with the different foci, was calculated for an area comprising the whole Y and Z extent of the insular cortex. The ALE was calculated across studies such that the summed ALE of all peaks of each study was normalized to unity to ensure that studies reporting large numbers of peaks cannot disproportionally dominate the resulting ALE maps. The average extent of the insular cortex was determined from the T1 multi-subject template provided with SPM5. The anatomical boundaries of the insula are described in the study by Makris et al. (2006). The procedure as described so far generates ALE maps for each of the modalities/categories investigated. Peaks that lay more than 5 mm outside the border of the insula were regarded as outliers and were excluded. The spatial union of all ALE values finally could be portrayed as an ALE-value map which differentially describes the reproducibility of an effect within different insular subregions. Statistical significance was assessed using a (non-parametric) single threshold permutation test as already described in previous works (Turkeltaub et al., 2002; Laird et al., 2005). ALE scores inferred from the distribution of activation foci reported in the included studies of one functional category was compared against a single critical ALE score derived from an empirical null distribution. This null distribution resulted from 10,000 permutations of an equal number of foci equal to the sample used to generate the ALE that were distributed randomly throughout the insular cortex. The threshold was set at *p* < 0.01. This means that only ALE-values that extend a critical ALE value corresponding to a significance level of α = 0.01 are indicated in the ALE maps.

### Definition of the domain general region (DGR)

The DGR in the AIC (red dashed circle in Figure 2) was defined based on the study of Dosenbach and colleagues who, based on mixed design fMRI experiments using 10 different tasks, found the bilateral anterior insula/frontal operculum region to show reliable start-cue and sustained activations across all or nearly all tasks (Dosenbach et al., 2006) which was interpreted as a representation of task sets. This generalized type of activation was found in the dorsal part of the AIC (black dot in Figure 2). It is clear that the DGR cannot be reduced to a single point corresponding to the reported peak coordinates. On the other hand, the exact size/spatial extent of the DRG is currently unclear. We defined the DGR large enough to encompass a peak related to meta-analytically defined, supramodal aesthetic appraisal (Brown et al., 2011) (back triangle in Figure 2A), as this supramodal processing may be an aspect of the domain general function defining the DGR. Our conclusions, however, do not critically depend on the size assigned to the DGR, because the peak reported by Dosenbach et al. (2006) directly falls into the region with empathy-related VBM effects (Figure 2B) and, therefore, an overlap between any DGR defined based on the study (Dosenbach et al., 2006) with our VBM effects exists in any case, independent of the exact radius assumed for the DGR.

## Results

**Figure 1:**
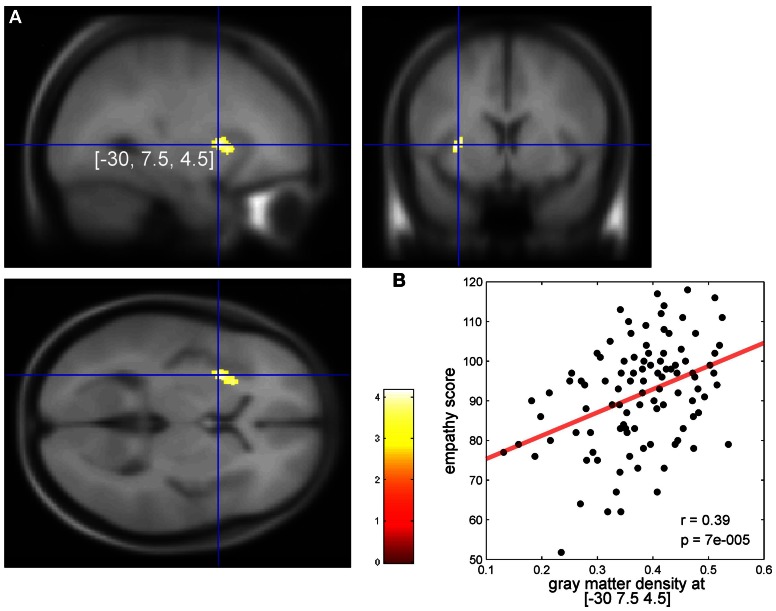
**(A)** Significant correlation in the left dorsal anterior insular cortex (AIC) between individual empathy scores in 101 healthy females and individual cortical gray values (*p* < 0.001). Axial slice at the (MNI) x/y/z coordinate [−30, 7.5, 4.5] (global, maximal correlation). *T*-values are color-coded. **(B)** Correlation of individual gray matter density (arbitrary units) and empathy scores at the peak coordinates from **(A)**. We report these correlations for descriptive purposes. Our conclusions are entirely based on the findings as shown in **(A)** because one has to keep in mind that the strength of correlation at peak coordinates as shown in **(B)** may overestimate the true effect (Vul et al., 2009).

Overall empathy scores (*M* = 91.6, *SD* = 13.3) of the E-Scale (Leibetseder et al., 2001) were normally distributed (Kolmogorov–Smirnov Test, *p* = 0.58). Means for the overall empathy score (*M* = 3.66, *SD* = 0.53) were similar to a previous study investigating a comparable sample of healthy individuals using the same questionnaire (*M* = 3.56, *SD* = 0.61). For more details see supplement of reference Krach et al. (2011). The VBM analysis revealed a single cluster of 185 voxels in the left AIC showed a significant positive correlation between individual empathy scores of the 101 subjects and GM density (*p* < 0.001, extent threshold *k* > 1.0 voxels, Figure 1). There were two peaks within this cluster at Montreal Neurologic Institute (MNI) x/y/z coordinates—30/7.5/4.5 (*T* = 4.17) and at—24/18/6 (*T* = 3.65).

Sixteen fMRI studies on empathy for pain (comprising 344 individuals, 191 females, 42 peaks, 23 in the left insula), 44 studies on emotion in healthy subjects (comprising 756 subjects and 46 peaks in the left insula) and 57 studies on physical pain in healthy individuals (comprising 690 subjects and 88 peaks in the left insula) fulfilled the inclusion criteria for the ALE meta-analyses (see Materials and Methods). Studies on pain predominantly used thermal heat stimuli (*n* = 29). Thirteen studies used PET and 44 studies used fMRI. Studies on emotion used PET (*N* = 13) or fMRI (*n* = 31). The studies included in the ALE analyses according to our inclusion criteria are listed in Tables A–C.

**Figure 2:**
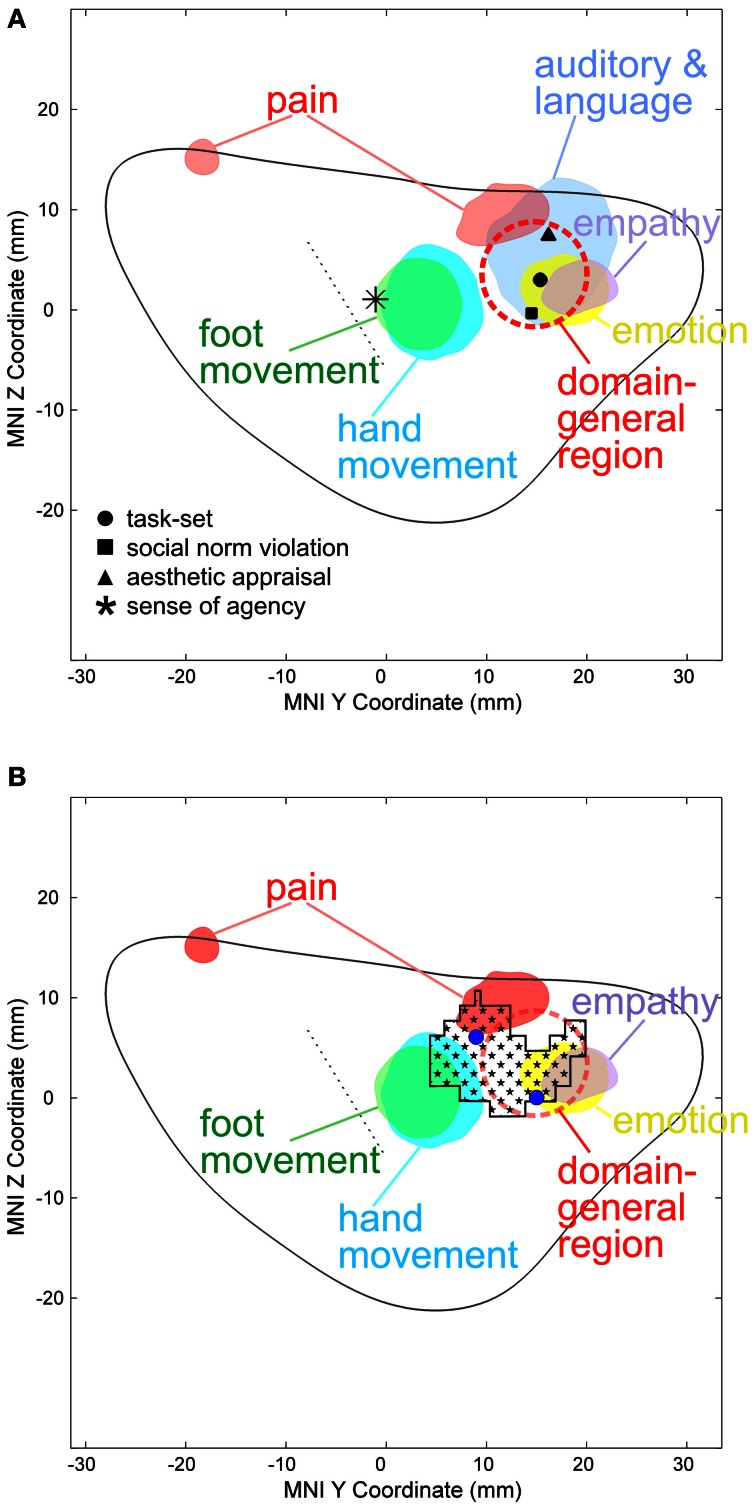
**(A)** Activation likelihood estimate (ALE) findings in the *left insula* and the MNI z coordinates (on the y-axis) and y coordinates (on the x-axis) related to the physical pain investigated with fMRI (red), emotion (yellow), empathy for pain (purple), hand (turquoise), and foot movement (green). The solid gray line indicates the mean outline of the left sagittal insula and the dashed gray line the central sulcus of the insula dividing the insula in an anterior and posterior part (see methods for more details). Importantly, studies on emotion in healthy individuals entering this ALE analysis did not include insula-coordinates which were related to the measurement of peripheral physiological changes. In a recent meta-analysis responses related to peripheral physiological changes resulting from emotional experiences were located in the ventral anterior insular cortex (Mutschler et al., 2009). Pain-related maximal ALE were found in the posterior insula (left) and in the dorsal anterior insula [left and right, see reference Mutschler et al. (2012) for more details]. Response peaks related to task-set processing based on conjoint analysis of data from 10 different fMRI tasks are indicated by red dashed circles [that is the approximate position of the domain-general region (DGR) of the insular cortex described in reference Dosenbach et al. (2006)], and peaks related to social norm violation (Sanfey et al., 2003), meta-analytically defined, supra-modal aesthetic appraisal (Brown et al., 2011), and sense of agency of hand movements (Farrer and Frith, 2002) by black squares, triangles, and star, respectively. Sense of agency of hand movements overlapped with sensorimotor processing. **(B)** Empathy-related voxel-based morphometric (VBM) findings in the *left insula* (indicated by black squares) overlapped with empathy and emotion and sensorimotor-related ALE, but also with the DGR. Blue dots represent empathy for pain-related coordinates from reference Singer et al. (2004).

In the left hemisphere, pain-related maximal ALE in healthy subjects were found in the posterior and dorsal mid-anterior insula, whereas in the right hemisphere maximal ALE was located in the dorsal mid-anterior insula. We further found that emotion-related maximal ALE in healthy individuals—excluding activation peaks in the insula related to the measurement of peripheral physiological changes—was located in the dorsal anterior insula. The maximal ALE related to studies investigating empathy for pain predominantly evoked activation in the dorsal part of the AIC. Figure 2 shows ALE findings.

## Discussion

Our results indicate regionally specific structural differences in the left dorsal AIC related to individual empathy scores in healthy individuals. GM density correlated in a confined region of the left dorsal AIC with empathy in healthy females. The insular region where we find structural differences overlaps with the peaks from a previous functional study correlating empathy-related responses fMRI with individual empathy scores (Singer et al., 2004), Figure 2B. This precise spatial correspondence strongly supports our VBM findings. Studies show that individuals with specialized abilities have markedly developed brain structures in specific regions related to their expertise (Kanai and Rees, 2011). For instance, an investigation by Maguire et al. on London taxi drivers found that GM measures derived from T1-weighted structural MRI are sensitive to experience. The authors showed that taxi drivers, with their knowledge of London's complex street plan, had enlarged posterior hippocampi in comparison to control subjects (Maguire et al., 2000). More recently, a longitudinal study demonstrates that learning to juggle increases GM density in occipito-parietal cortical areas in the adult brain involved in reaching and grasping (Scholz et al., 2009).

The results reveal that our morphometrically identified area in the dorsal AIC related to individual differences in empathy overlaps the DGR. A first indication toward a DGR was provided by Dosenbach et al. who conjointly analyzed mixed design fMRI experiments using 10 different tasks (Dosenbach et al., 2006). They found, among others, the bilateral anterior insula/frontal operculum region to show reliable start-cue and sustained activations across all or nearly all tasks. This generalized type of activation was found in the dorsal part of the AIC (black dot in Figure 2). The idea of a DGR was extended by Craig in his review on awareness and the AIC (Craig, 2009). The author observed that, in imaging studies, the AIC is reported to be activated in an astonishing number of studies from a broad range of topics including all types of subjective feelings, attention, cognitive choices, intentions, music, time perception, awareness of sensations, and movements, of visual and auditory percepts, of the trustworthiness of other individuals and concluded that “No other region of the brain is activated in all of these tasks.” In a subsequent meta-analysis of a wide range of functional imaging studies, the same region of the AIC that showed task-set-related responses in the study by Dosenbach et al. was also reliably activated by a wide range of auditory and language tasks as well as during social norm violation (Mutschler et al., 2009). Importantly, this study was restricted to studies which reported clear insular responses, i.e., ambiguous effects such as “insula/frontal operculum” were excluded, giving strong additional support for the existence of a domain-general region in the AIC. The idea of a DGR that is activated across nearly all kinds of tasks as well as Craig's observation that no other brain region shows such generalized responses was later also confirmed in a meta-analysis (Kurth et al., 2010). However, little is known regarding the exact location and the functional meaning of the DGR in the dorsal AIC. There is good evidence that the DGR is found in the dorsal anterior insula as indicated in Figure 2A. Importantly, compared to a previous interpretation (Brown et al., 2011) our ALE analysis shows that movement-related responses do not (or only to a very small degree) overlap with the domain-general area. The position of the main region with reproducible movement-related responses is located just posterior to the domain-general region (blue area in Figure 2). This movement-related area closely co-localized with insular peaks related to the sense of agency of hand movements (Mutschler et al., 2009). What is the functional meaning of the DGR—in particular for empathy? Several overarching functions have been proposed such as task-set processing (Dosenbach et al., 2006) and a role in awareness (Craig, 2009). Importantly, our findings—that the morphometrically identified area in the dorsal AIC related to individual differences in empathy overlaps the DGR—support the notion that its underlying neuronal substrate may be involved in integrating socio-emotional information during empathy.

We find that emotion-related responses in healthy subjects were preferentially located in the dorsal AIC. This region was distinct from the insular region associated with peripheral physiological changes resulting from emotional experiences and found that this activity was represented in the ventral AIC that was also the most likely site of insular co-activation with the amygdala (Mutschler et al., 2009). In the current study, insula-coordinates associated with emotion-related peripheral physiological changes were excluded. Therefore, our findings suggest that distinct functional insula regions may be involved in different aspects of emotional processing, whereby peripheral physiological correlates of emotional processing are mapped to the ventral anterior regions, while emotion-cognitive processes are mapped to a more dorsal-anterior region. More specifically, we assume that the dorsal anterior insula might play a pivotal role in integrating sensory stimuli with its salience, possibly via connections to the cingulate cortex. This interpretation is supported by the fact that in our study-sample subjects evaluated the emotional content of the presented stimuli and in direct agreement with recent findings showing that the dorsal anterior insula is more consistently involved in human cognition than ventral anterior and posterior networks (Chang et al., 2013), and the observed functional connectivity between the dorsal anterior insula and the dorsal anterior cingulate cortex, which that plays a crucial role in cognitive decision-making (Deen et al., 2011). Pain-related maximal ALE were found in the posterior insula and in the dorsal AIC. Both, emotion and pain related ALE findings are discussed in more detail elsewhere (Mutschler et al., 2012) because the aim of this meta-analysis was to relate them to studies on empathy. Recent neuroimaging studies show that the anterior insula and the anterior cingulate cortex are engaged during both, the experience and observation of pain (Singer et al., 2004; Lamm et al., 2011). It has been suggested that neural responses involved in both conditions might reflect a neuronal substrate which is related to the affective but not sensory aspect of pain (Singer et al., 2004). Together, our present ALE-findings support this notion and suggest that the posterior insula is involved in processing sensory aspect of pain, whereas the dorsal anterior insula is involved in emotion. In summary, we argue that the dorsal AIC plays a pivotal role in empathy (similarly as during emotion processing and pain) by integrating sensory stimuli with its salience, possibly via connections to the cingulate cortex. This assumption is also supported by the fact that ALE-findings related to emotion and empathy for pain and also the DGR—which has been associated with cognition—overlap in the dorsal anterior insula, suggesting that these functions share a common neural substrate (Dosenbach et al., 2006). As mentioned above we assume that the overall role of the morphometrically identified area in the dorsal AIC related to individual differences in empathy which overlaps the DGR might be involved in integrating information which is relevant for socio-emotional and cognitive processing. Thus, we assume that empathy is not (only) related to a specific “socio-emotional” interaction area, but to a superordinate “domain-general” area, in line with concepts of empathy that include not only social and emotional, but also cognitive aspects (Eisenberg and Miller, 1987; Decety and Jackson, 2004). Whether our findings in the dorsal AIC have also a relation to the “von Economo neurons” [VENs, (Von Economo, 1926)] remains to be determined. VENs have been hypothesized to play are role in social-emotional processing including empathy (Evrard et al., 2012; Seeley et al., 2012).

In the following paragraph potential limitations of this study are discussed and suggestions are made for future research. Similarly to previous imaging studies (e.g., Singer et al., 2004) we have investigated correlates of overall empathy in our study by using a widely applied and validated self-evaluation measure (Leibetseder et al., 2001, 2007). There is a potential concern about the influence of the *social desirability bias*—which refers to the tendency of subjects to answer self report items in a self-favoring manner—on the validity of questionnaire-based research (Edwards, 1957). It is discussed whether social desirability scales may be used to detect, minimize, and correct for social desirability bias in order to improve the validity of questionnaire-based research (e.g., Uziel, 2010). In a future study it would be interesting to measure empathy experimentally—e.g., by investigating the impact of compassion-based meditation on empathy (Mascaro et al., 2013)—and to relate functional activity to GM properties.

In this study, brain structure changes resulting from or predisposing to empathy have been investigated in a large sample of females because of increasing evidence for sex differences in empathy. Females score on average higher than males on self-report measures of empathy (Hoffman, 1977; Baron-Cohen and Wheelwright, 2004). Singer et al. observed in an fMRI study on empathy for pain that in males but not females empathetic reactions were absent for persons who were perceived as behaving unfairly (Singer et al., 2006). Recently, Van Honk et al. (2011) showed that the administration of the androgen hormone testosterone—which represents the largest hormonal difference between the sexes—impaired cognitive empathy in healthy females. As mentioned, the insular region where we find structural differences exactly overlaps with the peaks from a previous functional study correlating empathy-related responses fMRI with individual empathy scores (Singer et al., 2004). Notably, in this study also only females were investigated. A recent study found in a sample with mixed gender individual differences in trait empathy dimensions correlating with morphological differences in several brain areas including the anterior cingulated cortex and the right dorsolateral prefrontal cortex (Banissy et al., 2012). More specifically, based on an analysis with ROIs around peaks from previous functional studies on empathy, Banissy et al. report structural changes in the *ventral-most part of the insular cortex*, several centimeters apart from the area characterized in the present study. Interpretational difficulties however arise because (1) the peak at MNI coordinates −39, 9, −21 as reported by Banissy et al. (that was used to define the ventral insular ROI) is pain-related and not empathy-related as was assumed by the authors (see Appendix to Singer et al., 2004) and (2) a second, empathic concern-related peak reported to be in the insular region by Banissy et al. at MNI coordinates −48, 6, 18 is according to the probabilistic assignment obtained from the SPM Anatomy Toolbox (Eickhoff et al., 2005) located in Brodmann Area 44. In this study, structural changes specific to males or females were however not addressed and only changes that were consistent across the whole (mixed) sample were reported which may possibly explain the different results of their and our study. Alternatively, the different results between both studies may also be explained by the fact that in our study the focus was on global empathy whereas in the study by Banissy et al. on the relationship between components of empathy (empathic concern, personal distress, perspective taking, and fantasizing) and brain structure using a different measure. Because empathy crucially requires high-level integration of emotional, cognitive, and social components as well as of behavioral control the goal of our study was not to investigate different components of empathy but to explore the neural substrate that may underlay its neural integration. Our interpretation—that the dorsal AIC where we find structural differences depending on individual empathy might play a crucial role in modulating the efficiency of neural integration underlying emotional, cognitive, and sensorimotor information which is essential for global empathy—is in agreement with previous studies reporting that reduced GM volume in the AIC was associated with a lack of empathy in neuropsychiatric disorders such as in conduct disorder (Sterzer et al., 2007) and in psychopathy (de Oliveira-Souza et al., 2008). Further, as mentioned above, a recent study in combat veterans with traumatic brain injury shows that lesions in several brain regions, particularly in the insula, was associated with deficits in empathy (Driscoll et al., 2012). In future research based on larger samples it would be interesting to investigate the role of empathy subscales such as cognitive and emotional components (Leibetseder et al., 2007), and their relation to functional and structural data. The emotional component of empathy has been for instance closely linked to activation in the inferior frontal gyrus (Shamay-Tsoory et al., 2009; Banissy et al., 2012).

Finally, longitudinal studies are needed to clarify whether the empathy-related structural effects in the dorsal AIC that we find are due to a pre-existing brain characteristic or to empathy-experience, or whether it indicates both.

In addition, all neuroimaging studies entering our meta-analysis reported that only healthy individuals free of any neurological and psychiatric disorders were investigated. However, for future meta-analyses it would be important that neuroimaging studies specify more clearly the procedure regarding how they assessed and excluded individuals with neurological and psychiatric disorders [e.g., whether a structured clinical interview for Diagnostic and Statistical Manual IV (DSM-IV) axis I and axis II personality disorders was used]. Furthermore, it is also important to note that our meta-analysis on empathy included females and males. An interesting topic for future meta-analyses would be to examine whether there are functional differences between male and female samples with respect to empathy. Finally, future meta-analyses should also investigate whether the dorsal AIC is rather involved in empathy for pain (Singer et al., 2004) and social rejection (Eisenberger et al., 2003) than in empathy for positive emotions and if yes why this could be the case. Only few brain imaging studies to date have examined empathy for positive emotions (Jabbi et al., 2007; Mobbs et al., 2009; Morelli et al., 2012), therefore ALE meta-analysis could not be performed. If the dorsal AIC is related to empathy and is essential for high-level integration, it should be activated during empathy for all types of emotions. However, the few studies on empathy for positive emotions suggest that the dorsal AIC might not be involved (Mobbs et al., 2009; Morelli et al., 2012), but a meta-analytic analysis based on a large sample of studies would be required to resolve this issue. In future it would be also important to meta-analytically examine studies on empathy for negative emotions which show insula activation, such as empathy for disgust (Wicker et al., 2003), embarrassment (Krach et al., 2011), social rejection (Masten et al., 2011), and anxiety (Morelli et al., 2012), as well as studies on functional components of empathy.

In summary, the dorsal AIC where we find structural differences depending on individual empathy may be key in modulating the efficiency of neural integration underlying emotional, cognitive, and sensorimotor information which is essential for empathy. Furthermore, our results support a functional subdivision of the human insula in functionally distinct regions. These include the ventral anterior insula which is involved in mapping peripheral physiological information during emotional experiences and the dorsal AIC which plays a crucial role in integrating sensory stimuli with salience possibly via connections to the cingulate cortex. The dorsal anterior insula constitutes an auditory and language area and the mid anterior insula plays a pivotal role in sensorimotor processing. Finally, the posterior insula may be involved in processing sensory aspects of nociceptive information and the dorsal AIC may have an integrative role during emotional-cognitive evaluation of a noxious stimuli and the associated sensorimotor response. Together, these findings provide new important insights into the functional organization of the human insular cortex in healthy individuals, and the functional map may be helpful for understanding dysfunction in conditions affecting empathy such as borderline personality disorder with co-morbid posttraumatic stress disorder, autistic spectrum disorders, psychopathy/antisocial personality disorder, conduct disorder, and schizophrenia.

## Funding

We gratefully acknowledge financial support by the Swiss National Science Foundation (SNSF fellowship to Isabella Mutschler).

### Conflict of interest statement

The authors declare that the research was conducted in the absence of any commercial or financial relationships that could be construed as a potential conflict of interest.

## Appendix

**Table A:** **Sixteen fMRI imaging studies reporting anterior insula activity related to empathy for pain (comprising 344 individuals, 191 females, and 42 peaks, 23 left and 19 right)**.

**References**	**Method**	**Handedness**	**Smoothing (FWHM)**	**Investigated effect and sample size**	**Coordinates x/y/z Talairach (Tal) or MNI space**	
Akitsuki and Decety, 2009	fMRI	Right	6	Animated visual images (hands and feet) depicting painful vs. non-painful situations, *N* = 26 (14 females)	−26/22/8 (MNI)	40/24/0 (MNI)
					−38/−2/14 (MNI)	38/0/12 (MNI)
					−40/−4/−6 (MNI)	
Botvinick et al., 2005	fMRI	Left	12	Videos of painful vs. neutral facial expressions, *N* = 12 females	−42/18/2 (Tal)	53/0/−5 (Tal)
Cheng et al., 2007	fMRI	Right	8	Animated visual images (hands, feet, mouth) depicting painful situations (acupuncture needles) vs. non-painful situations (Q-tips) or fixation, *N* = 28 (14 females)	−36/16/−1 (MNI)	46/16/−2 (MNI)
						40/20/−9 (MNI)
Cheng et al., 2010	fMRI	Right	8	Animated visual images (hands and feet) depicting painful vs. non-painful situations, *N* = 36 (18 females)	−40/12/−6 (MNI)	42/10/−8 (MNI)
Danziger et al., 2009	fMRI	Left	8	Pictures (hands and feet) of painful vs. non-painful situations and painful vs. neutral facial expressions, *N* = 13 (7 females)	−48/9/6 (MNI)	36/6/6 (MNI)
					−39/0/9 (MNI)	42/−9/−6 (MNI)
					−39/12/−6 (MNI)	
Decety et al., 2010	fMRI	Right	6	Painful vs. neutral facial expressions (videos), *N* = 22 (11 females)	−26/30/0 (MNI)	36/24/−8 (MNI)
					−40/26/−4 (MNI)	
					−26/26/−10 (MNI)	
Gu et al., 2010	fMRI	Right	8	Pictures (hands and feet) of painful vs. non-painful situations, *N* = 18 (9 females)	−34/20/2 (MNI)	−
					−38/−2/0 (MNI)	
Han et al., 2009	fMRI	Right	8	Videos of painful vs. neutral facial expressions, *N* = 22 (12 females)	−36/24/2 (MNI)	−
Jackson et al., 2006	fMRI	Right	6	Pictures (hands and feet) of painful vs. non-painful situations, *N* = 34 (20 females)	−30/18/3 (MNI)	33/21/0 (MNI)
Lamm et al., 2007	fMRI	Right	6	Videos of painful facial expressions vs. baseline (exact coordinates for this contrast not published in the paper but asked from the author), *N* = 17 (8 females)	−32/24/−8 (MNI)	34/20/8 (MNI)
					−32/24/2 (MNI)	
Lamm et al., 2010	fMRI	Right	6	Pictures (hands and arms) of painful (needles) vs. non-painful (Q-tips) situations (exact coordinates for this contrast not published in the paper but asked from the author), *N* = 24 (12 females)	−34/24/−12 (MNI)	40/21/2 (MNI)
Moriguchi et al., 2007	fMRI	Right	6	Pictures (hands and feet) of painful vs. non-painful situations, *N* = 14 (12 females)	−40/−10/0 (MNI)	−
Ogino et al., 2007	fMRI	Right	7,6	Pictures of painful situations vs. rest, *N* = 10 (no females)	−	40/8/−8 (MNI)
						36/−4/12 (MNI)
Singer et al., 2004	fMRI	Right	10	Observing pain in others (loved person) vs. observing no pain, *N* = 16 (16 females)	−36/12/0 (MNI)	39/12/3 (MNI)
					−45/30/−3 (MNI)	60/15/3 (MNI)
Singer et al., 2006	fMRI	Left	10	Observing pain in others (fair persons) vs. observing no pain, *N* = 32 (16 females)	−33/33/3 (MNI)	39/−6/12 (MNI)
Singer et al., 2008	fMRI	Right	10	Observing pain in others (loved person) vs. observing no pain, *N* = 20 (no females)	−	30/27/6 (MNI)
						45/21/0 (MNI)

**Table B:** **Fifty-seven neuroimaging studies investigating pain in healthy subjects entered the ALE meta-analyses**.

**References**	**Method**	**Handedness**	**Smoothing (FWHM)**	**Investigated effect (modality), sample size, body site of stimulation**	**Coordinates x/y/z Talairach (Tal) or MNI space**	
**Albuquerque et al., 2006**	**fMRI**	**Right**	**8.6**	**Thermal pain (heat) vs. warm stimuli, ***N*** = 8, right**	**–**	**28.3/3.4/12.4 (Tal)**
Becerra et al., 2001	fMRI	Right	6	Thermal pain (heat) vs. warm stimuli, *N* = 8, left	−46/−9/6 (Tal)	34/−6/9 (Tal)
					–43/−18/18 (Tal)	31/18/12 (Tal)
						34/6/9 (Tal)
**Bingel et al., 2003**	**fMRI**	**Right**	**6**	**Painful laser stimuli vs. rest, ***N*** = 14, right**	**−39/6/0 (MNI)**	**39/0/9 (MNI)**
					**−36/0/12 (MNI)**	**36/6/9 (MNI)**
Bingel et al., 2007	fMRI	Right	8	Thermal pain (heat) vs. rest, *N* = 20, left	−36/6/6 (MNI)	39/9/9 (MNI)
Brooks et al., 2002	fMRI	Mixed	9	Thermal pain (heat) vs. warm stimuli, *N* = 18, right	−30/24/7 (Tal)	36/18/5 (Tal)
					−39/−20/20 (Tal)	42/−17/20 (Tal)
					−36/23/−4 (Tal)	42/20/−6 (Tal)
					−36/12/2 (Tal)	
**Brooks et al., 2005**	**fMRI**	**Right**	**8.6**	**Thermal pain (heat) vs. rest, ***N*** = 14, right**	**−32/22/0 (MNI)**	**38/16/0 (MNI)**
					**−36/12/0 (MNI)**	**38/12/2 (MNI)**
					**−38/−16/8 (MNI)**	**38/14/0 (MNI)**
					**−34/22/−4 (MNI)**	**38/16/2 (MNI)**
					**−40/8/−2 (MNI)**	**40/12/−4 (MNI)**
					**−38/−18/8 (MNI)**	
					**−36/24/−6 (MNI)**	
					**−38/4/4 (MNI)**	
					**−38/−10/10 (MNI)**	
**Carlsson et al., 2006**	**fMRI**	**Right**	**12**	**Electrical pain vs. non-painful electrical stimulation, ***N*** = 10, right**	**−34/10/−4 (MNI)**	**36/12/6 (MNI)**
					**−40/−18/−6 (MNI)**	**46/−16/0 (MNI)**
Casey et al., 2001	PET	Right	9	Thermal pain (heat) vs. warm stimuli, *N* = 14, left	−35/3/4 (Tal)	33/23/7 (Tal)
						39/1/11 (Tal)
**Christmann et al., 2007**	**fMRI**	**Right**	**8.6**	**Electrical pain vs. rest, ***N*** = 6, right**	**−40/−18/15 (Tal)**	**41/10/3 (Tal)**
					**−31/17/12 (Tal)**	**41/5/0 (Tal)**
Cole et al., 2010	fMRI	Mixed	4	Mechanical pain vs. non-painful mechanical stimulation, *N* = 15, right	−42/2/6 (MNI)	44/14/−6 (MNI)
					−40/−6/−2 (MNI)	40/18/−4 (MNI)
Derbyshire et al., 1997	PET	Mixed	10	Painful vs. non-painful laser stimuli, *N* = 12, right	−30/−2/4 (Tal)	−
Derbyshire and Osborn, 2009	fMRI	Right	6	Thermal pain (heat) vs. warm stimuli, *N* = 12, mixed	−	40/−20/2 (MNI)
						42/24/14 (MNI)
Derbyshire and Jones, 1998	PET	Mixed	8	Thermal pain (heat) vs. warm stimuli, *N* = 12, right	−	38/8/−4 (Tal)
Dube et al., 2009	fMRI	Mixed	8	Thermal pain (heat) vs. warm stimuli, *N* = 12, left	−32/28/−8 (MNI)	36/8/12 (MNI)
					−40/0/−12 (MNI)	36/20/−4 (MNI)
					−36/−12/0 (MNI)	
Ducreux et al., 2006	fMRI	Mixed	6	Thermal pain (cold) vs. rest, *N* = 6, right	−36/−6/12 (MNI)	40/14/−2 (MNI)
					−30/20/−2 (MNI)	
**Frankenstein et al., 2001**	**fMRI**	**Right**	**8**	**Thermal pain (cold) vs. rest, ***N*** = 12, right**	**–**	**40/0/18 (MNI)**
						**34/12/12 (MNI)**
Freund et al., 2007	fMRI	Right	8	Electrical pain vs. rest, *N* = 15, mixed	−40/−16/16 (Tal)	40/14/−6 (Tal)
Gracely et al., 2002	fMRI	Right	8.6	Mechanical pain vs. non-painful mechanical stimulation, *N* = 16, left	−48/12/−2 (Tal)	38/4/0 (Tal)
Gundel et al., 2008	fMRI	Right	8	Thermal pain (heat) vs. rest, *N* = 12, left	−27/18/9 (MNI)	27/15/−3 (MNI)
						39/−18/15 (MNI)
Hofbauer et al., 2004	PET	Right	14	Thermal pain (heat) vs. warm stimuli, *N* = 15, left	−	29/13/13 (Tal)
**Kong et al., 2006**	**fMRI**	**Right**	**8**	**Thermal pain (heat): high vs. low pain intensity, ***N*** = 16, right**	**−36/−16/14 (MNI)**	**36/−20/16 (MNI)**
**Kurata et al., 2002**	**fMRI**	**Right**	**8.6**	**Thermal pain (heat) vs. warm stimuli, ***N*** = 5, right**	**−39/5/10 (Tal)**	**40/−6/15 (Tal)**
					**−43/−11/13 (Tal)**	
					**−41/−7/6 (Tal)**	
Laureys et al., 2002	PET	Mixed	8.6	Electrical pain vs. rest, *N* = 15, mixed	−40/−16/10 (MNI)	32/−10/−2 (MNI)
de Leeuw et al., 2006a	fMRI	Right	8.6	Thermal pain (heat) vs. warm stimuli, *N* = 9, left	−34/10/7 (Tal)	35/10/8 (Tal)
de Leeuw et al., 2006b	fMRI	Right	8.6	Thermal pain (heat) vs. warm stimuli, *N* = 9, left	−34/10/7 (Tal)	35/10/8 (Tal)
Lorenz et al., 2002	PET	Right	9	Thermal pain (heat) vs. rest, *N* = 14, left	−39/17/7 (Tal)	35/12/2 (Tal)
**Lorenz et al., 2008**	**fMRI**	**Right**	**8**	**Mechanical pain vs. rest, ***N*** = 22, right**	**−40/−4/−2 (MNI)**	**42/10/−8 (MNI)**
**Lui et al., 2008**	**fMRI**	**Right**	**8 × 8 × 10**	**Mechanical pain vs. rest and mechanical pain vs. non-painful mechanical stimulation, ***N*** = 14, right**	**−40/8/−4 (MNI)**	**–**
					**−44/−8/0 (MNI)**	
					**−56/0/4 (MNI)**	
					**−40/4/−4 (MNI)**	
					**−60/4/4 (MNI)**	
Maihofner and Handwerker, 2005	fMRI	Right	4	Thermal pain (heat) vs. warm stimuli, *N* = 12, left	−32/18/9 (Tal)	39/−16/9 (Tal)
					−38/−11/9 (Tal)	
Maihofner et al., 2006	fMRI	Mixed	4	Mechanical pain vs. rest and thermal pain (heat) vs. warm stimuli, *N* = 14, right	−28/19/12 (Tal)	35/17/9 (Tal)
					−46/13/8 (Tal)	38/11/11 (Tal)
					−46/13/8 (Tal)	37/−22/15 (Tal)
Maihofner et al., 2007	fMRI	Mixed	4	Mechanical pain vs. rest, *N* = 14, left	−43.8/13.2/11.3 (Tal)	36.7/15.4/3 (Tal)
Mobascher et al., 2009a	fMRI	Right	6	Painful laser stimuli vs. rest, *N* = 20, left	−36/22/0 (MNI)	38/22/−2 (MNI)
Mobascher et al., 2009b	fMRI	Right	6	Painful laser stimuli vs. rest, *N* = 12, left	−34/−20/14 (MNI)	40/4/6 (MNI)
Mochizuki et al., 2007	fMRI	Right	8	Thermal pain (cold) vs. non-painful cold stimuli, *N* = 14, left	−42/10/−4 (MNI)	36/10/6 (MNI)
**Nemoto et al., 2003**	**PET**	**Right**	**16**	**Painful laser stimuli vs. rest, ***N*** = 12, right**	**−34/−6/12 (MNI)**	**42/−2/6 (MNI)**
Ochsner et al., 2006	fMRI	Mixed	6	Thermal pain (heat) vs. warm stimuli, *N* = 13, right	−	42/0/10 (MNI)
						38/−22/12 (MNI)
Paulson et al., 1998	PET	Right	9	Thermal pain (heat) vs. warm stimuli, *N* = 10, left	−30/−6/14 (MNI)	33/1/0 (MNI)
						39/−22/16 (MNI)
Peyron et al., 2007	fMRI	Right	10	Electrical pain vs. rest, *N* = 9, left	−	54/18/−14 (MNI)
Ploghaus et al., 2001	fMRI	Right	8	Thermal pain (heat), high vs. low pain intensity, *N* = 8, left	−39/−19/15 (Tal)	−
Ploner et al., 2010	fMRI	Mixed	8	Painful vs. non-painful laser stimulation, *N* = 16, right	−48/4/−2 (MNI)	30/22/10 (MNI)
					−44/−4/10 (MNI)	34/−16/8 (MNI)
Rolls et al., 2003	fMRI	Right	7	Mechanical pain vs. rest, *N* = 9, left	−44/−2/10 (MNI)	36/0/8 (MNI)
					−60/−32/18 (MNI)	
Schmahl et al., 2006	fMRI	Mixed	8	Thermal pain (heat) vs. warm stimuli, *N* = 12, right	−33/14/10 (MNI)	33/17/13 (MNI)
					−30/15/10 (MNI)	33/15/10 (MNI)
**Seifert and Maihofner, 2007**	**fMRI**	**Right**	**4**	**Thermal pain (cold) vs. non-painful cold stimuli, ***N*** = 12, right**	**−31/13/9 (Tal)**	**38/19/7 (Tal)**
					**−39/−17/16 (Tal)**	
**Stammler et al., 2008**	**fMRI**	**Right**	**7**	**Mechanical pain vs. rest, ***N*** = 12, right**	**−33/18/15 (Tal)**	**38/−5/14 (Tal)**
					**−36/−6/11 (Tal)**	
Straube et al., 2009	fMRI	Right	8	Electrical pain vs. rest, *N* = 18, left	−39/14/7 (Tal)	43/17/4 (Tal)
					−36/11/10 (Tal)	40/8/7 (Tal)
					−39/11/10 (Tal)	34/11/4 (Tal)
					−33/20/7 (Tal)	41/14/7 (Tal)
**Svensson et al., 1998**	**PET**	**Right**	**12**	**Thermal pain (heat) vs. rest and high vs. low pain intensity, ***N*** = 10, right**	**−43/5/2 (Tal)**	**29/10/11 (Tal)**
					**−46/1/2 (Tal)**	**37/−1/15 (Tal)**
					**−38/5/11 (Tal)**	
**Symonds et al., 2006**	**fMRI**	**Right**	**3**	**Electrical pain vs. rest, ***N*** = 9, right**	**–**	**42/−5/2 (Tal)**
						**35/14/2 (Tal)**
						**44/−8/−6 (Tal)**
**Tolle et al., 1999**	**PET**	**Right**	**18**	**Thermal pain (heat) vs. warm stimuli, ***N*** = 12, right**	**−35/−6/7 (Tal)**	**–**
**Tseng et al., 2010**	**fMRI**	**Right**	**8**	**Thermal pain (heat) vs. warm stimuli, ***N*** = 12, right**	**−36/−20/6 (MNI)**	**38/−2/6 (MNI)**
					**−34/16/6 (MNI)**	**36/16/2 (MNI)**
**Valet et al., 2004**	**fMRI**	**Right**	**8**	**Thermal pain (heat) vs. warm stimuli, ***N*** = 7, right**	**−40/−16/14 (MNI)**	**34/−14/10 (MNI)**
					**−40/6/6 (MNI)**	**38/16/−4 (MNI)**
Vanhaudenhuyse et al., 2009	fMRI	Mixed	8	Painful laser stimuli vs. rest, *N* = 13, left	−60/−34/10 (MNI)	40/−14/14 (MNI)
Vogt et al., 1996	PET	Right	20	Thermal pain (heat) vs. warm stimuli, *N* = 7, left	−34/10/−4 (Tal)	36/0/4 (Tal)
von Leupoldt et al., 2009	fMRI	Mixed	6	Thermal pain (heat), high vs. low pain intensity, *N* = 14, mixed	−39/6/6 (MNI)	42/9/0 (MNI)
					−42/6/−6 (MNI)	36/0/−10 (MNI)
**Wagner et al., 2007**	**PET**	**Right**	**12**	**Thermal pain (heat) vs. warm stimuli, ***N*** = 7, right**	**–**	**30/18/10 (MNI)**
						**40/4/−8 (MNI)**
**Watson et al., 2009**	**fMRI**	**Right**	**5**	**Painful vs. non-painful laser stimuli, ***N*** = 11, right**	**−34/8/12 (MNI)**	**40/−14/10 (MNI)**
Xu et al., 1997	PET	Right	8.6	Painful laser stimuli vs. rest, *N* = 6, left	−38/12/12 (Tal)	36/−6/0/ (Tal)
						32/16/−4 (Tal)
Ziv et al., 2010	fMRI	Mixed	6	Thermal pain (heat) vs. warm stimuli, *N* = 10, mixed	−	38/13/11 (MNI)

Total number of peaks 175 (88 peaks in the left and 87 peaks in the right insula). Number of investigated individuals: 690. Studies on pain predominantly used thermal heat stimuli (n = 29). Thirteen studies applied positron emission tomography (PET) and 44 studies functional magnetic resonance imaging (fMRI). Twenty studies investigated pain in right-handed subjects by stimulating the right body side (highlighted in bold, total number of peaks: 66 peaks, 37 in the left and 29 peaks in the right insula).

**Table C:** **Fourty-four imaging studies on emotional processing in healthy subjects**.

**References**	**Method**	**Handedness**	**Smoothing (FWHM)**	**Investigated effect and sample size**	**Coordinates x/y/z Talairach (Tal) or MNI space**	
Anand et al., 2005	fMRI	Mixed	8	Watching negative vs. neutral pictures, *N* = 15	−44/−11/14 (Tal)	46/−7/12 (Tal)
Bartels and Zeki, 2000	fMRI	Mixed	10	Watching photos of a loved one vs. neutral person, *N* = 17	−44/6/−4 (Tal)	44/10/−6 (Tal)
Bartels and Zeki, 2004	fMRI	Mixed	10	Watching photos of own vs. other child, *N* = 20	−42/8/−4 (Tal)	−
Blood and Zatorre, 2001	PET	Mixed	8.7	Listening to subject-selected music (positive valance) vs. neutral control condition, *N* = 10	−39/12/11 (Tal)	32/15/3 (Tal)
Britton et al., 2005	PET	Right	12	Imagery of emotionally evocative vs. neutral autobiographic events, *N* = 14	−32/−8/8 (MNI)	32/4/12 (MNI)
Britton et al., 2006	fMRI	Right	6	Watching negative (anger) vs. neutral pictures (IAPS), *N* = 12	−42/24/6 (MNI)	−
Calder et al., 2007	PET	Right	8	Watching negative (disgust) vs. neutral pictures, *N* = 14	−	32/18/8 (MNI)
Damasio et al., 2000	PET	Right	6	Remembering negative vs. neutral personal life episodes, *N* = 39	−35/12/−9 (Tal)	42/21/0 (Tal)
					−36/−1/13 (Tal)	36/0/13 (Tal)
					−37/13/−4 (Tal)	36/11/2 (Tal)
					−41/−3/12 (Tal)	33/3/6 (Tal)
						46/1/0 (Tal)
Donix et al., 2010	fMRI	Right	10	Personal (affect-laden) memory vs. neutral control condition, *N* = 15	−32/6/−6 (MNI)	50/18/−10 (MNI)
Ethofer et al., 2009	fMRI	Right	10	Listening to angrily vs. neutrally spoken words (prosody), parallel task to classify words, *N* = 24	−24/15/−18 (MNI)	30/6/−18 (MNI)
Eugene et al., 2003	fMRI	Right	12	Negative (sadness) vs. neutral films, *N* = 20	−39/15/2 (Tal)	36/3/−3 (Tal)
Fink et al., 1996	PET	Right	8.7	Personal (affect-laden) memory vs. neutral control condition, *N* = 7	−	28/18/−4 (Tal)
Fitzgerald et al., 2004	fMRI	Right	6	Remembering negative life event (disgust) vs. non-emotional event, *N* = 12	−34/6/6 (MNI)	40/−12/0 (MNI)
					−42/−4/−6 (MNI)	
Garrett and Maddock, 2006	fMRI	Right	4	Watching negative vs. neutral pictures, *N* = 9	−	38/11/−4 (MNI)
George et al., 1996	PET	Mixed	8.7	Remembering sad vs. neutral life events (triggered by sad and neutral faces), *N* = 10	−42/12/4 (Tal)	−
					−28/−14/12 (Tal)	
					−34/10/−12 (Tal)	
Gundel et al., 2003	fMRI	Mixed	8	Watching pictures of a dead person (grief) vs. neutral control condition, *N* = 8	−	38/18/−6 (Tal)
Hofer et al., 2006	fMRI	Right	8	Watching and classifying negative vs. neutral pictures, *N* = 19	−	36/24/4 (Tal)
Hutcherson et al., 2005	fMRI	Right	3.75	Watching and rating amusing vs. neutral films, *N* = 28	−	44/−10/−5 (Tal)
						42/−3/−7 (Tal)
						44/9/3 (Tal)
Immordino-Yang et al., 2009	fMRI	Right	8.7	Remembering stories that elicit admiration/compassion vs. neutral control condition, parallel rating of emotional states, *N* = 13	−27/20/7 (Tal)	39/5/4 (Tal)
					−27/17/7 (Tal)	30/14/7 (Tal)
					−42/8/4 (Tal)	39/−1/−2 (Tal)
						39/5/4 (Tal)
Klucken et al., 2009	fMRI	Right	9	Watching negative vs. neutral pictures, *N* = 32	−36/21/−3 (MNI)	30/18/−18 (MNI)
Kotz et al., 2003	fMRI	Right	8.7	Rating emotionally vs. neutrally spoken words (prosody), *N* = 12	−28/22/0 (Tal)	−
					−29/22/0 (Tal)	
Lane et al., 1997	PET	Right	8.7	Remembering sad vs. neutral life episodes, *N* = 12	−36/6/4 (Tal)	−
Levesque et al., 2003	fMRI	Right	12	Watching sad vs. neutral films, *N* = 20	−39/15/−1 (Tal)	−
Levesque et al., 2003	fMRI	Right	12	Watching sad vs. neutral films, *N* = 20	−39/15/−1 (Tal)	−
Liotti et al., 2000	PET	Right	9.9	Remembering negative vs. neutral life episodes, *N* = 8	−23/2/10 (Tal)	34/−22/18 (Tal)
						44/15/−3 (Tal)
Marci et al., 2007	PET	Right	10	Remembering negative (anger) vs. neutral life episodes, *N* = 10	−28/−18/12 (MNI)	−
Mathews et al., 2004	fMRI	Right	8	Watching negative (fear) vs. neutral pictures, *N* = 24	−34/−2/14 (MNI)	−
Moll et al., 2002	fMRI	Right	6	Watching negative vs. neutral pictures (IAPS), *N* = 7	−	30/27/7 (Tal)
Moll et al., 2005	fMRI	Right	8.7	Reading sentences that elicit indignation vs. neutral control condition, *N* = 13	−32/−15/0 (Tal)	−
Noriuchi et al., 2008	fMRI	Right	8	Watching silent videos of own vs. other child, *N* = 13	−	36/24/6 (MNI)
Reiman et al., 1997	PET	Right	8.7	Remembering affect-laden vs. neutral life events, *N* = 12	−34/18/0 (Tal)	44/14/0 (Tal)
Schafer et al., 2005	fMRI	Right	8	Watching negative vs. neutral pictures, *N* = 40	−36/−3/−12 (MNI)	30/12/−18 (MNI)
					−36/15/−15 (MNI)	33/15/−15 (MNI)
					−36/12/−15 (MNI)	
Schienle et al., 2002	fMRI	Right	9	Watching negative vs. neutral pictures, *N* = 12	−42/9/−15 (MNI)	42/18/−12 (MNI)
					−27/21/−12 (MNI)	
Shapira et al., 2003	fMRI	Right	8.7	Watching negative (disgust) vs. neutral pictures (IAPS), *N* = 8	−36/11/6 (Tal)	33/12/6 (Tal)
Stark et al., 2007	fMRI	Right	9	Watching and rating negative (disgust) vs. neutral pictures, *N* = 66	−36/0/−12 (MNI)	−
Straube et al., 2010	fMRI	Right	8	Watching threatening vs. neutral films, *N* = 40	−33/17/10 (Tal)	39/20/13 (Tal)
Takahashi et al., 2006	fMRI	Right	8	Reading sentences that elicit jealousy vs. neutral control condition, *N* = 11	−42/4/12 (MNI)	−
Taylor et al., 2000	PET	Mixed	13	Watching negative vs. neutral pictures, *N* = 14	−28/19/2 (Tal)	−
Taylor et al., 2003	PET	Mixed	14	Watching negative vs. neutral pictures, *N* = 10	−33/−8/2 (Tal)	−
Teasdale et al., 1999	fMRI	Right	7.2	Watching/reading positive vs. neutral pictures and sentences, *N* = 6	−38/−6/4 (Tal)	40/−17/4 (Tal)
Van Dillen et al., 2009	fMRI	Right	6	Watching negative vs. neutral pictures (IAPS), *N* = 17	−31/−1/−9 (Tal)	31/−3/−9 (Tal)
Waugh et al., 2008	fMRI	Mixed	8	Watching negative vs. neutral pictures (IAPS), *N* = 30	−38/0/3 (MNI)	38/9/−9 (MNI)
Wright et al., 2004	fMRI	Mixed	8.7	Watching negative (disgust) vs. neutral pictures (IAPS), *N* = 8	−	31/20/2 (Tal)
Zald and Pardo, 2002	PET	Right	10	Listening to unpleasant vs. neutral sounds, *N* = 8	−	33/−4/−4 (Tal)
						35/−10/−4 (Tal)
Zeki and Romaya, 2008	fMRI	Mixed	9	Watching photos of a hated vs. neutral person, *N* = 17	−48/9/0 (MNI)	51/12/−6 (MNI)

Number of investigated individuals: 756. Total number of peaks: 89 (46 peaks in the left and 43 peaks in the right insula). Sixty-seven activation peaks resulted from negative emotional states contrasted against a neutral condition (37 in the left and 30 peaks in the right insula). Seventeen activation peaks resulted from positive emotional states contrasted against a neutral condition (8 peaks in the left and 9 peaks in the right insula). Five activation peaks resulted from contrasting emotional states of mixed valence against a neutral control. Thirteen studies applied positron emission tomography (PET) and 31 studies functional magnetic resonance imaging (fMRI).

## References

[B1] Ashburner J. (2007). A fast diffeomorphic image registration algorithm. Neuroimage 38, 95–113 10.1016/j.neuroimage.2007.07.007 17761438

[B2] Banissy M. J. Kanai R. Walsh V. Rees G. (2012). Inter-individual differences in empathy are reflected in human brain structure. Neuroimage 62, 2034–2039 10.1016/j.neuroimage.2012.05.081 22683384 PMC3778747

[B3] Baron-Cohen S. Wheelwright S. (2004). The empathy quotient: an investigation of adults with Asperger syndrome or high functioning autism, and normal sex differences. J. Autism Dev. Disord. 34, 163–175 15162935 10.1023/b:jadd.0000022607.19833.00

[B4] Bora E. Gokcen S. Veznedaroglu B. (2008). Empathic abilities in people with schizophrenia. Psychiatry Res. 160, 23–29 10.1016/j.psychres.2007.05.017 18514324

[B5] Borsook D. Edwards R. Elman I. Becerra L. Levine J. (2013). Pain and analgesia: the value of salience circuits. Prog. Neurobiol. [Epub ahead of print]. 10.1016/j.pneurobio.2013.02.003 23499729 PMC3644802

[B6] Brown S. Gao X. Tisdelle L. Eickhoff S. B. Liotti M. (2011). Naturalizing aesthetics: brain areas for aesthetic appraisal across sensory modalities. Neuroimage 58, 250–258 10.1016/j.neuroimage.2011.06.012 21699987 PMC8005853

[B7] Cauda F. D'Agata F. Sacco K. Duca S. Geminiani G. Vercelli A. (2011). Functional connectivity of the insula in the resting brain. Neuroimage 55, 8–23 10.1016/j.neuroimage.2010.11.049 21111053

[B8] Chang L. J. Yarkoni T. Khaw M. W. Sanfey A. G. (2013). Decoding the role of the insula in human cognition: functional parcellation and large-scale reverse inference. Cereb. Cortex 23, 739–749 10.1093/cercor/bhs065 22437053 PMC3563343

[B9] Craig A. D. (2009). How do you feel–now? The anterior insula and human awareness. Nat. Rev. Neurosci. 10, 59–70 10.1038/nrn2555 19096369

[B10] Davis M. H. (1983). Measuring individual-differences in empathy—evidence for a multidimensional approach. J. Pers. Soc. Psychol. 44, 113–126

[B11] Decety J. Jackson P. L. (2004). The functional architecture of human empathy. Behav. Cogn. Neurosci. Rev. 3, 71–100 10.1177/1534582304267187 15537986

[B12] Decety J. Moriguchi Y. (2007). The empathic brain and its dysfunction in psychiatric populations: implications for intervention across different clinical conditions. Biopsychosoc. Med. 1:22 10.1186/1751-0759-1-22 18021398 PMC2206036

[B13] Deen B. Pitskel N. B. Pelphrey K. A. (2011). Three systems of insular functional connectivity identified with cluster analysis. Cereb. Cortex 21, 1498–1506 10.1093/cercor/bhq186 21097516 PMC3116731

[B14] de Oliveira-Souza R. Hare R. D. Bramati I. E. Garrido G. J. Azevedo Ignacio F. Tovar-Moll F. (2008). Psychopathy as a disorder of the moral brain: fronto-temporo-limbic grey matter reductions demonstrated by voxel-based morphometry. Neuroimage 40, 1202–1213 10.1016/j.neuroimage.2007.12.054 18289882

[B15] Dosenbach N. U. Visscher K. M. Palmer E. D. Miezin F. M. Wenger K. K. Kang H. C. (2006). A core system for the implementation of task sets. Neuron 50, 799–812 10.1016/j.neuron.2006.04.031 16731517 PMC3621133

[B16] Driscoll D. M. Dal Monte O. Solomon J. Krueger F. Grafman J. (2012). Empathic deficits in combat veterans with traumatic brain injury: a voxel-based lesion-symptom mapping study. Cogn. Behav. Neurol. 25, 160–166 10.1097/WNN.0b013e318280cf4e 23277137

[B17] Dymond R. F. (1949). A scale for the measurement of empathic ability. J. Consult. Psychol. 13, 127–133 18118267 10.1037/h0061728

[B18] Edwards A. L. (1957). The Social Desirability Variable in Personality Assessment and Research. New York, NY: Dryden

[B19] Eickhoff S. B. Stephan K. E. Mohlberg H. Grefkes C. Fink G. R. Amunts K. (2005). A new SPM toolbox for combining probabilistic cytoarchitectonic maps and functional imaging data. Neuroimage 25, 1325–1335 10.1016/j.neuroimage.2004.12.034 15850749

[B20] Eisenberg N. Miller P. A. (1987). The relation of empathy to prosocial and related behaviors. Psychol. Bull. 101, 91–119 10.1037/0033-2909.101.1.91 3562705

[B21] Eisenberger N. I. Lieberman M. D. Williams K. D. (2003). Does rejection hurt? An fMRI study of social exclusion. Science 302, 290–292 10.1126/science.1089134 14551436

[B22] Evrard H. C. Forro T. Logothetis N. K. (2012). Von economo neurons in the anterior insula of the macaque monkey. Neuron 74, 482–489 10.1016/j.neuron.2012.03.003 22578500

[B23] Farrer C. Frith C. D. (2002). Experiencing oneself vs another person as being the cause of an action: the neural correlates of the experience of agency. Neuroimage 15, 596–603 10.1006/nimg.2001.1009 11848702

[B24] Goleman D. (1998). Working with Emotional Intelligence. New York, NY: Bantam Books

[B25] Herringa R. Phillips M. Almeida J. Insana S. Germain A. (2012). Post-traumatic stress symptoms correlate with smaller subgenual cingulate, caudate, and insula volumes in unmedicated combat veterans. Psychiatry Res. 203, 139–145 10.1016/j.pscychresns.2012.02.005 23021615 PMC3466380

[B26] Heuser M. Thomann P. A. Essig M. Bachmann S. Schroder J. (2011). Neurological signs and morphological cerebral changes in schizophrenia: an analysis of NSS subscales in patients with first episode psychosis. Psychiatry Res. 192, 69–76 10.1016/j.pscychresns.2010.11.009 21498055

[B27] Hoffman M. L. (1977). Sex differences in empathy and related behaviors. Psychol. Bull. 84, 712–722 897032

[B28] Jabbi M. Swart M. Keysers C. (2007). Empathy for positive and negative emotions in the gustatory cortex. Neuroimage 34, 1744–1753 10.1016/j.neuroimage.2006.10.032 17175173

[B29] Johansen-Berg H. (2012). The future of functionally-related structural change assessment. Neuroimage 62, 1293–1298 10.1016/j.neuroimage.2011.10.073 22056531 PMC3677804

[B30] Kanai R. Rees G. (2011). The structural basis of inter-individual differences in human behaviour and cognition. Nat. Rev. Neurosci. 12, 231–242 10.1038/nrn3000 21407245

[B31] Kim J. H. Suh S. I. Seol H. Y. Oh K. Seo W. K. Yu S. W. (2008). Regional grey matter changes in patients with migraine: a voxel-based morphometry study. Cephalalgia 28, 598–604 10.1111/j.1468-2982.2008.01550.x 18422725

[B32] Krach S. Cohrs J. C. de Echeverria Loebell N. C. Kircher T. Sommer J. Jansen A. (2011). Your flaws are my pain: linking empathy to vicarious embarrassment. PLoS ONE 6:e18675 10.1371/journal.pone.0018675 21533250 PMC3076433

[B33] Kurth F. Zilles K. Fox P. T. Laird A. R. Eickhoff S. B. (2010). A link between the systems: functional differentiation and integration within the human insula revealed by meta-analysis. Brain Struct. Funct. 214, 519–534 10.1007/s00429-010-0255-z 20512376 PMC4801482

[B34] Labate A. Cerasa A. Gambardella A. Aguglia U. Quattrone A. (2008). Hippocampal and thalamic atrophy in mild temporal lobe epilepsy: a VBM study. Neurology 71, 1094–1101 10.1212/01.wnl.0000326898.05099.04 18824674

[B35] Laird A. R. Fox P. M. Price C. J. Glahn D. C. Uecker A. M. Lancaster J. L. (2005). ALE meta-analysis: controlling the false discovery rate and performing statistical contrasts. Hum. Brain Mapp. 25, 155–164 10.1002/hbm.20136 15846811 PMC6871747

[B36] Lamm C. Decety J. Singer T. (2011). Meta-analytic evidence for common and distinct neural networks associated with directly experienced pain and empathy for pain. Neuroimage 54, 2492–2502 10.1016/j.neuroimage.2010.10.014 20946964

[B37] Leibetseder M. Laireiter A. R. Koller T. (2007). Structural analysis of the E-scale. Pers. Individ. Dif. 42, 547–561

[B38] Leibetseder M. Laireiter A.-R. Riepler A. (2001). E-Scale: questionnaire for the assessment of empathy in fictitious as well as real-life situations—Description and psychometric properties. Zeitschrift für Differentielle und Diagnostische Psychologie 22, 70–85

[B39] Maguire E. A. Gadian D. G. Johnsrude I. S. Good C. D. Ashburner J. Frackowiak R. S. J. (2000). Navigation-related structural change in the hippocampi of taxi drivers. Proc. Natl. Acad. Sci. U.S.A. 97, 4398–4403 10.1073/pnas.070039597 10716738 PMC18253

[B40] Makris N. Goldstein J. M. Kennedy D. Hodge S. M. Caviness V. S. Faraone S. V. (2006). Decreased volume of left and total anterior insular lobule in schizophrenia. Schizophr. Res. 83, 155–171 10.1016/j.schres.2005.11.020 16448806

[B41] Marangoni C. Garcia S. Ickes W. Teng G. (1995). Empathic accuracy in a clinically relevant setting. J. Pers. Soc. Psychol. 68, 854–869 10.1037/0022-3514.68.5.854 7776183

[B42] Mascaro J. S. Rilling J. K. Tenzin Negi L. Raison C. L. (2013). Compassion meditation enhances empathic accuracy and related neural activity. Soc. Cogn. Affect. Neurosci. 8, 48–55 10.1093/scan/nss095 22956676 PMC3541495

[B43] Masten C. L. Morelli S. A. Eisenberger N. I. (2011). An fMRI investigation of empathy for ‘social pain’ and subsequent prosocial behavior. Neuroimage 55, 381–388 10.1016/j.neuroimage.2010.11.060 21122817

[B44] Mehrabian A. Epstein N. (1972). A measure of emotional empathy. J. Pers. 40, 525–543 4642390 10.1111/j.1467-6494.1972.tb00078.x

[B45] Mesulam M. M. Mufson E. J. (1982). Insula of the old world monkey. III: efferent cortical output and comments on function. J. Comp. Neurol. 212, 38–52 10.1002/cne.902120104 7174907

[B46] Miller P. A. Eisenberg N. (1988). The relation of empathy to aggressive and externalizing/antisocial behavior. Psychol. Bull. 103, 324–344 10.1037/0033-2909.103.3.324 3289071

[B47] Mobbs D. Yu R. Meyer M. Passamonti L. Seymour B. Calder A. J. (2009). A key role for similarity in vicarious reward. Science 324, 900 10.1126/science.1170539 19443777 PMC2839480

[B48] Morelli S. A. Rameson L. T. Lieberman M. D. (2012). The neural components of empathy: predicting daily prosocial behavior. Soc. Cogn. Affect. Neurosci. [Epub ahead of print]. 10.1093/scan/nss088 22887480 PMC3871722

[B49] Mufson E. J. Mesulam M. M. (1982). Insula of the old world monkey. II: afferent cortical input and comments on the claustrum. J. Comp. Neurol. 212, 23–37 10.1002/cne.902120103 7174906

[B50] Mutschler I. Ball T. Wankerl J. Strigo I. A. (2012). Pain and emotion in the insular cortex: evidence for functional reorganization in major depression. Neurosci. Lett. 520, 204–209 10.1016/j.neulet.2012.03.095 22503725

[B51] Mutschler I. Schulze-Bonhage A. Glauche V. Demandt E. Speck O. Ball T. (2007). A rapid sound-action association effect in human insular cortex. PLoS ONE 2:e259 10.1371/journal.pone.0000259 17327919 PMC1800344

[B52] Mutschler I. Wieckhorst B. Kowalevski S. Derix J. Wentlandt J. Schulze-Bonhage A. (2009). Functional organization of the human anterior insular cortex. Neurosci. Lett. 457, 66–70 10.1016/j.neulet.2009.03.101 19429164

[B53] Nieuwenhuys R. (2012). The insular cortex: a review. Prog. Brain Res. 195, 123–163 10.1016/B978-0-444-53860-4.00007-6 22230626

[B54a] Oldfield R. C. (1971). The assessment and analysis of handedness: the Edinburgh inventory. Neuropsychologia 9, 97–113 5146491 10.1016/0028-3932(71)90067-4

[B54] Peters J. Dauvermann M. Mette C. Platen P. Franke J. Hinrichs T. (2009). Voxel-based morphometry reveals an association between aerobic capacity and grey matter density in the right anterior insula. Neuroscience 163, 1102–1108 10.1016/j.neuroscience.2009.07.030 19628025

[B55] Premack D. Woodruf G. (1978). Does the chimpanzee have a theory of mind? Behav. Brain Sci. 1, 515–526

[B56] Preston S. D. (2007). A perception-action model for empathy, in Empathy in Mental Illness, eds Farrow T. Woordruff P. (Cambridge: Cambridge University Press), 428–447

[B57] Roepke S. Vater A. Preissler S. Heekeren H. R. Dziobek I. (2012). Social cognition in borderline personality disorder. Front. Neurosci. 6:195 10.3389/fnins.2012.00195 23335877 PMC3543980

[B58] Sanfey A. G. Rilling J. K. Aronson J. A. Nystrom L. E. Cohen J. D. (2003). The neural basis of economic decision-making in the Ultimatum Game. Science 300, 1755–1758 10.1126/science.1082976 12805551

[B59] Scholz J. Klein M. C. Behrens T. E. Johansen-Berg H. (2009). Training induces changes in white-matter architecture. Nat. Neurosci. 12, 1370–1371 10.1038/nn.2412 19820707 PMC2770457

[B60] Seeley W. W. Merkle F. T. Gaus S. E. Craig A. D. Allman J. M. Hof P. R. (2012). Distinctive neurons of the anterior cingulate and frontoinsular cortex: a historical perspective. Cereb. Cortex 22, 245–250 10.1093/cercor/bhr005 21653703

[B61] Shamay-Tsoory S. G. Aharon-Peretz J. Perry D. (2009). Two systems for empathy: a double dissociation between emotional and cognitive empathy in inferior frontal gyrus versus ventromedial prefrontal lesions. Brain 132, 617–627 10.1093/brain/awn279 18971202

[B62] Shirtcliff E. A. Vitacco M. J. Graf A. R. Gostisha A. J. Merz J. L. Zahn-Waxler C. (2009). Neurobiology of empathy and callousness: implications for the development of antisocial behavior. Behav. Sci. Law 27, 137–171 10.1002/bsl.862 19319834 PMC2729461

[B63] Singer T. Seymour B. O'Doherty J. Kaube H. Dolan R. J. Frith C. D. (2004). Empathy for pain involves the affective but not sensory components of pain. Science 303, 1157–1162 10.1126/science.1093535 14976305

[B64] Singer T. Seymour B. O'Doherty J. P. Stephan K. E. Dolan R. J. Frith C. D. (2006). Empathic neural responses are modulated by the perceived fairness of others. Nature 439, 466–469 10.1038/nature04271 16421576 PMC2636868

[B65] Sterzer P. Stadler C. Poustka F. Kleinschmidt A. (2007). A structural neural deficit in adolescents with conduct disorder and its association with lack of empathy. Neuroimage 37, 335–342 10.1016/j.neuroimage.2007.04.043 17553706

[B66] Stotland E. Kenneth E. Mathews J. Sherman S. E. Hansson R. O. Richardson B. (1978). Empathy, Fantasy and Helping. (Beverly Hills: Sage Library of Social Research), 27–45

[B67] Touroutoglou A. Hollenbeck M. Dickerson B. C. Feldman Barrett L. (2012). Dissociable large-scale networks anchored in the right anterior insula subserve affective experience and attention. Neuroimage 60, 1947–1958 10.1016/j.neuroimage.2012.02.012 22361166 PMC3345941

[B68] Turkeltaub P. E. Eden G. F. Jones K. M. Zeffiro T. A. (2002). Meta-analysis of the functional neuroanatomy of single-word reading: method and validation. Neuroimage 16, 765–780 10.1006/nimg.2002.1131 12169260

[B69] Uziel L. (2010). Rethinking social desirability scales: from impression management to interpersonally oriented self-control. Perspect. Psychol. Sci. 5, 243–262 10.1177/1745691610369465 26162157

[B70] Van Honk J. Schutter D. J. Bos P. A. Kruijt A. W. Lentjes E. G. Baron-Cohen S. (2011). Testosterone administration impairs cognitive empathy in women depending on second-to-fourth digit ratio. Proc. Natl. Acad. Sci. U.S.A. 108, 3448–3452 10.1073/pnas.1011891108 21300863 PMC3044405

[B71] Von Economo C. (1926). Eine neue Art Spezialzellen des Lobus cinguli and Lobus insulae. Zeitschrift für die gesamte Neurologie und Psychiatrie 100, 706–712

[B72] Vul E. Harris C. Winkielman P. Pashler H. (2009). Puzzlingly high correlations in fmri studies of emotion, personality, and social cognition. Perspect. Psychol. Sci. 4, 274–290 10.1111/j.1745-6924.2009.01125.x 26158964

[B73] Wicker B. Keysers C. Plailly J. Royet J. P. Gallese V. Rizzolatti G. (2003). Both of us disgusted in My Insula: the common neural basis of seeing and feeling disgust. Neuron 40, 655–664 10.1016/S0896-6273(03)00679-2 14642287

[B74] Wiech K. Lin C. S. Brodersen K. H. Bingel U. Ploner M. Tracey I. (2010). Anterior insula integrates information about salience into perceptual decisions about pain. J. Neurosci. 30, 16324–16331 10.1523/JNEUROSCI.2087-10.2010 21123578 PMC6634837

[B75] Zaki J. Ochsner K. (2012). The neuroscience of empathy: progress, pitfalls and promise. Nat. Neurosci. 15, 675–680 10.1038/nn.3085 22504346

